# Single-site laparoscopic right hemicolectomy for acute cecal volvulus: a case report

**DOI:** 10.1186/s40792-016-0179-9

**Published:** 2016-05-26

**Authors:** Yoshinori Kagawa, Takeshi Kato, Atsushi Naito, Yoshihiro Morimoto, Yasufumi Sato, Ryuichi Kuwahara, Tomo Ishida, Yasuo Oneda, Kohei Murakami, Junichi Inatome, Yoshiteru Katsura, Yoshiaki Ohmura, Atsushi Takeno, Chiyomi Egawa, Yutaka Takeda, Shigeyuki Tamura

**Affiliations:** Department of Surgery, Kansai Rosai Hospital, Amagasaki, Hyogo Japan

**Keywords:** Cecal volvulus, Single-site laparoscopic surgery, Decompression, Appendix, Catheterization, Cecum, Laparoscopic surgery, Laparoscopic colectomy, Right hemicolectomy, Bowel obstruction

## Abstract

**Background:**

Cecal volvulus is an uncommon cause of acute abdomen in patients. Cecal volvulus is currently treated mostly with right hemicolectomy with laparotomy, which is an invasive surgical procedure. Less invasive techniques, such as endoscopic decompression, have a poor success rate.

**Case presentation:**

We report a case of cecal volvulus in a 35-year-old male patient. He was successfully treated with single-site laparoscopic decompression by inserting a catheter through the amputated appendix, detorsion, and hemicolectomy. This approach was less invasive than the traditional approach and resulted in satisfactory outcomes and cosmesis.

**Conclusions:**

Application of single-site laparoscopic colectomy to acute cecal volvulus is feasible using decompression of the dilated colon by inserting a catheter through the amputated appendix. To the best of our knowledge, this is the first report of this treatment.

## Background

Cecal volvulus involves torsion of the flexible cecum, causing bowel obstruction, ischemia, necrosis, and perforation [1]. This is an uncommon cause of acute abdomen [2]. Cecal volvulus presents with abdominal pain, vomiting, and abdominal distension. The success rate of endoscopic decompression is only 15–20 %, and therefore, an emergency operation is necessary [3]. The preferred surgical procedure for cecal volvulus is right hemicolectomy with laparotomy [4]. We report here a case of a patient with cecal volvulus who was successfully treated with single-site laparoscopic decompression by inserting a catheter through the amputated appendix, detorsion, and right hemicolectomy.

## Case presentation

**Fig. 1:**
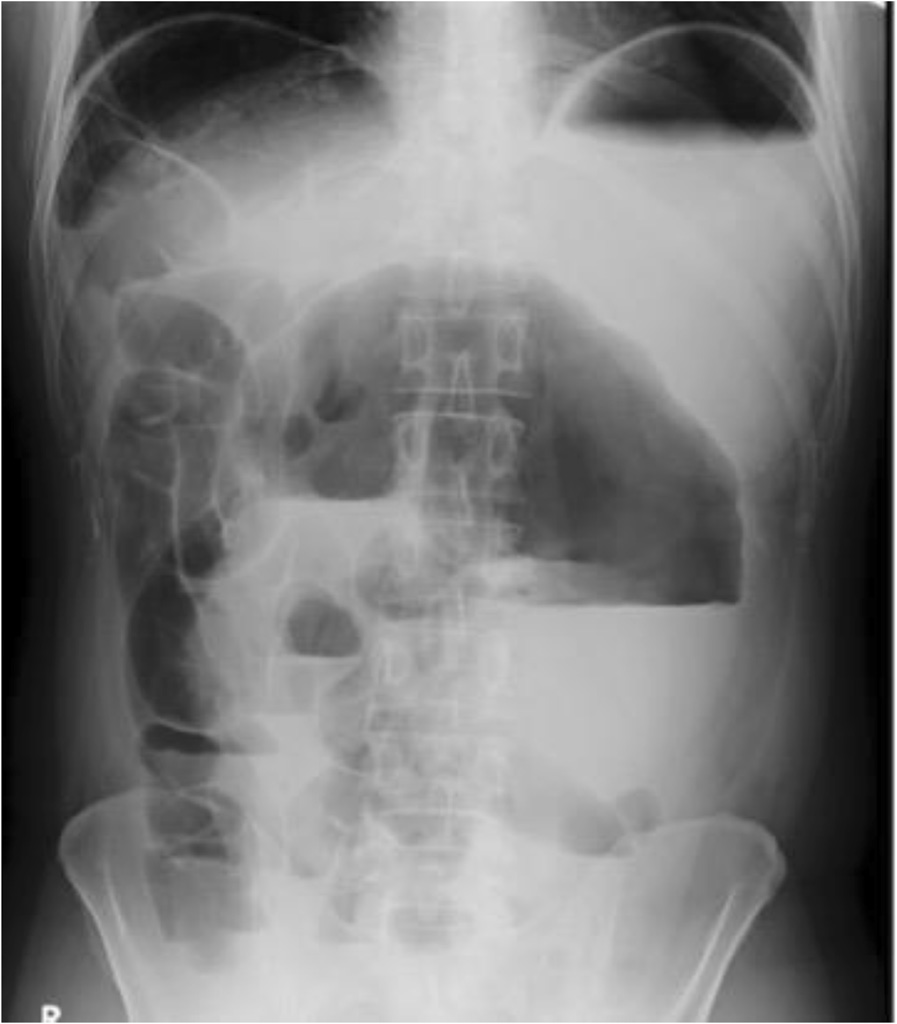
Upright plain abdominal X-ray showing a hugely dilated large bowel loop and distended small bowel with air fluid levels

**Fig. 2:**
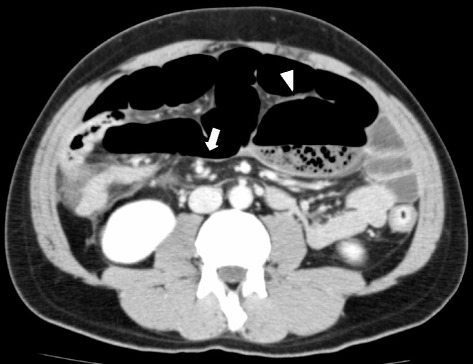
Computed tomography shows a “whirl” sign (*arrow*) and distended cecum (*arrowhead*) in the upper left abdominal cavity and small bowel

A 35-year-old man was admitted to a previous hospital with acute abdominal pain, vomiting, and abdominal distension. He was diagnosed with ileus based on an abdominal X-ray (Fig. 1) and computed tomography findings (Fig. 2). He underwent medical treatment with a long intestinal tube to decompress the small bowel distention. Because his condition was not improved, even after 2 days, the attending doctor consulted with the authors about surgical treatment. The patient was transferred to our hospital for emergency surgery. He had no history of abdominal disease. On examination, he was febrile with abdominal distension and rebound tenderness. Blood tests showed that the white blood cell count was 15,400 cells/mm^3^, the neutrophil fraction was 84.8 %, and the C-reactive protein level was 245.72 nmol/L. An abdominal computed tomography scan showed twisting of the mesentery around the ileocolic vessels (whirl sign) and a massively dilated cecum with associated small bowel dilation (Fig. 2). We diagnosed him with ileus due to cecal volvulus and decided to perform an emergency operation.

**Fig. 3:**
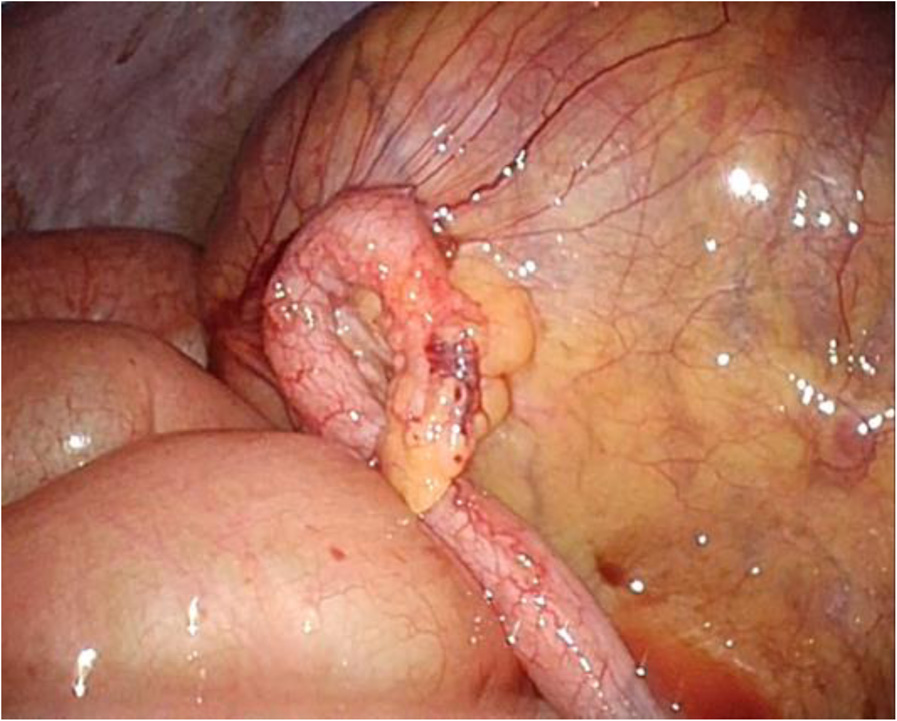
Laparoscopic view of the upper mid abdomen showing a distended cecum due to rotation of the right colon

**Fig. 4:**
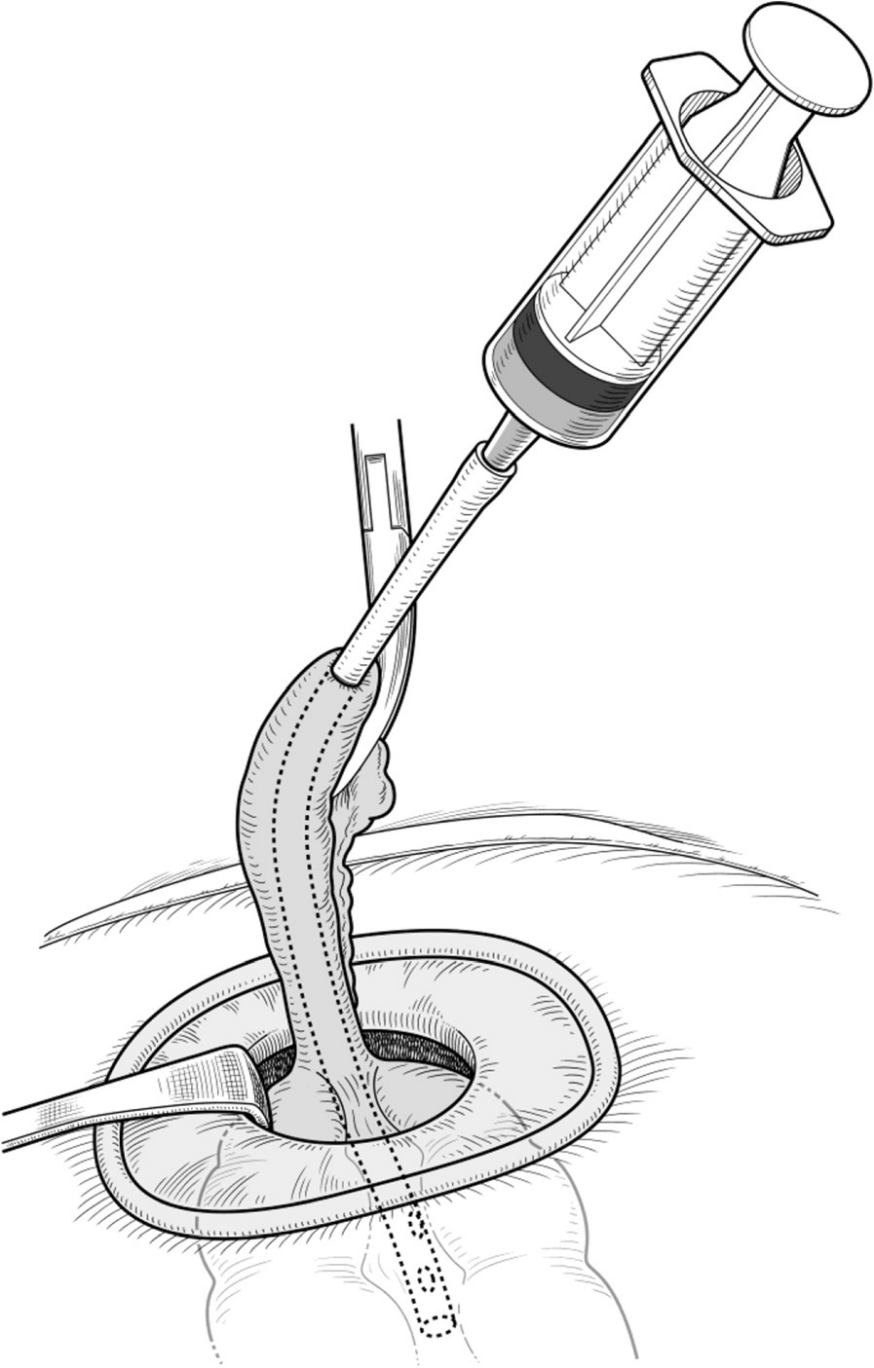
Decompression of a dilated large bowel. The mobilized appendix was extracted through the umbilical incision. A Nelaton catheter was inserted through the appendix into the cecum under direct vision. The air and contents were aspirated

**Fig. 5:**
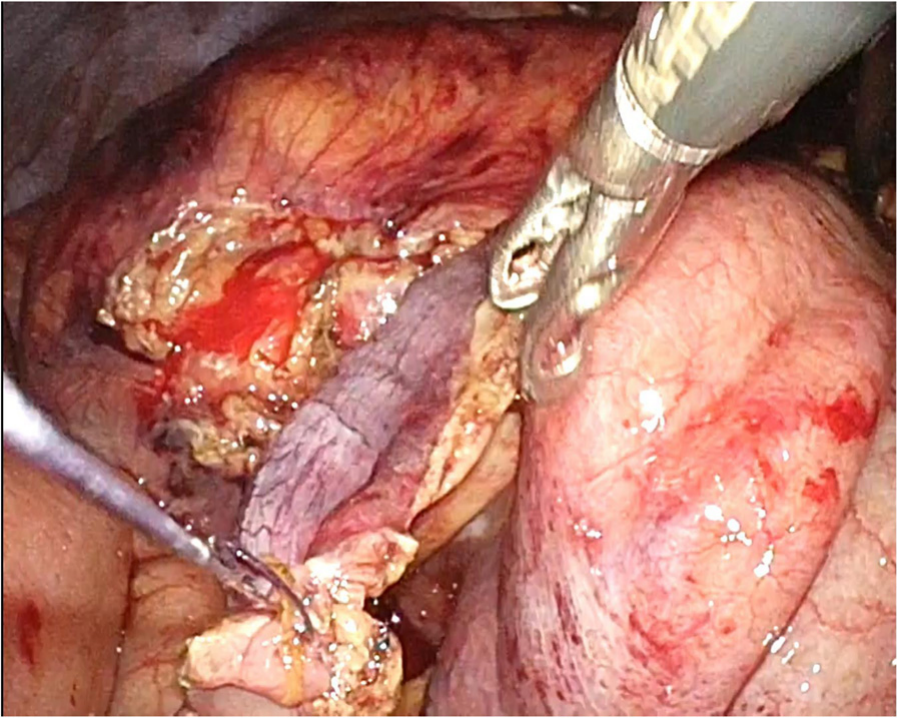
Decompressed cecum after aspiration through the appendix. The appendix was ligated at the proximal site of the tubing

**Fig. 6:**
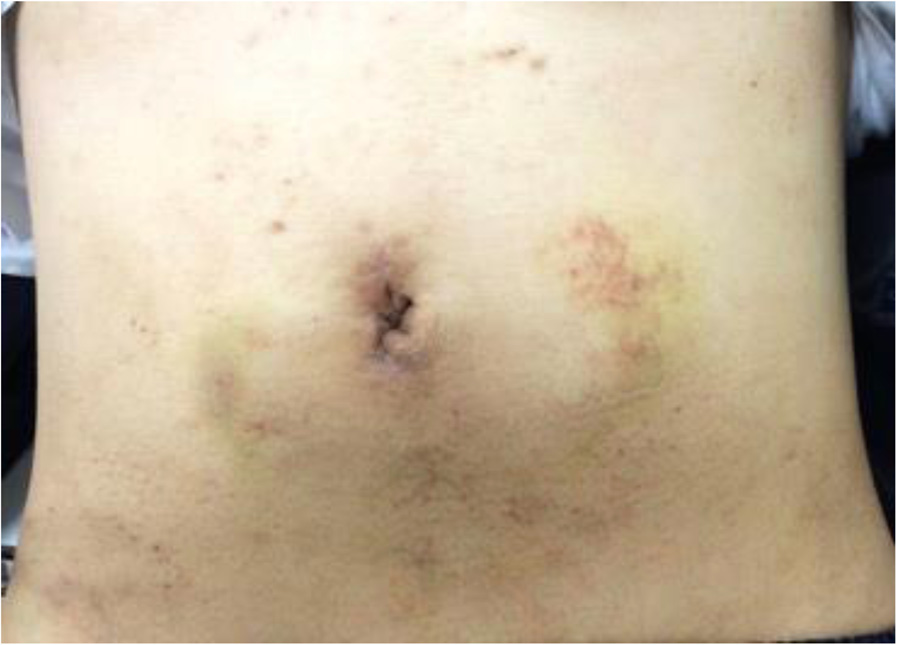
Cosmetic abdominal view after single-site laparoscopic colectomy for acute cecal volvulus on day 14

In the operation room, the patient was placed in the Trendelenburg position under general anesthesia. The main monitor was placed on the right side of the patient at approximately the level of the shoulder. A secondary monitor was placed on the right side of the patient at the same level. Surgery was started with a transumbilical 3-cm incision using the Hasson technique. A small wound retractor (Lap-Protector, Hakko, Nagano, Japan) and a single port device (EZ Access, Hakko, Nagano, Japan) were used. Three 5-mm trocars (EZ Trocar, Hakko, Nagano, Japan) were mounted on a LAP-Protector, and a 5-mm flexible endoscope (Olympus, Tokyo, Japan) was used. After pneumoperitoneum was induced, an extremely dilated cecum was observed in the left and right upper quadrants. The small bowel was dilated, and the root of the appendix was observed on the left side with the apex of the appendix pulled up to the right side (Fig. 3). The twisting point could not be visualized because of the distended and tense cecum. Returning the twisted cecum to the restricted abdominal space occupied by the tense and dilated large and small bowels was technically difficult. Decompression was needed to create a favorable situation. Using the appendix as an access route to the cecum, we dissected the mesoappendix using a surgical energy device (Thunderbeat, Olympus, Tokyo, Japan) and then mobilized the appendix. The appendix was pulled out through the umbilical incision to the outside of the abdomen. The tip of the appendix was amputated, and a 14 Fr Nelaton catheter tube (Terumo Corporation, Tokyo, Japan) was inserted through the appendix into the cecum under direct vision [5]. The catheter tube was connected to a 50-ml syringe or a suction tube, and then the air and contents were aspirated (Fig. 4). The appendix was tied at the proximal side of the tubing site after aspiration. We observed the abdominal cavity again and the distended colon was decompressed (Fig. 5). Some parts of the cecum showed ischemic change. At this point, we decided that right hemicolectomy was required in this case. The rotated colon was returned to a more normal position. The ascending colon was mobilized from the retroperitoneum, similar to the procedure for single-site laparoscopic right hemicolectomy for colon cancer. The ileocecal artery and vein were divided intraperitoneally using a surgical energy device. The cecum and terminal ileum were externalized through the umbilical incision. A right hemicolectomy was performed with side-to-side anastomosis of the terminal ileum to the transverse colon with interrupted 3-0 Vicryl (Ethicon, Somerville, NJ, USA). The umbilical scar was closed with 0 Vicryl Plus (Ethicon). The operation time was 187 min, and blood loss was 79 g. The patient made an uneventful and early recovery. He could walk around without any analgesic agents from postoperative day 1. Oral intake was started on postoperative day 3, and he was discharged on postoperative day 7. The patient returned to normal life in 2 weeks. The small umbilical incision healed with no morbidity. The cosmetic umbilical scar is shown in Fig. 6. By using the single laparoscopic approach, the patient recovered earlier with less pain and the smaller incision was healed more cosmetically.

### Discussion

The incidence of cecal volvulus ranges from 2.8 to 7.1 per million people per year [1, 2]. Cecal volvulus is responsible for 1–1.5 % of all adult intestinal obstructions [6] and 25–40 % of all cases of volvulus involving the colon [7–9]. Cecal volvulus can occur when the ascending colon, cecum, and terminal ileum are abnormally mobile, which represents a failure of complete rotation, descent, and fixation of the right colon.

Clinical presentation of acute cecal volvulus is related to features of proximal large bowel obstruction. The symptoms of cecal volvulus are colicky pain and abdominal diffuse distention [7, 10]. Abdominal X-rays show marked distension of the large bowel from the right lower quadrant to the epigastrium or left upper quadrant. Computed tomography scans also show a massively dilated cecum with associated small bowel dilation. Computed tomography confirms the diagnosis of cecal volvulus in approximately 90 % of patients, with the remainder diagnosed at the time of the operation [11, 12].

Colonoscopic decompression is generally not recommended in the initial treatment of cecal volvulus because the success rate is only approximately 30 % [3], and there is a potential for perforation and delayed surgical management. Surgical management is usually required for acute cecal volvulus because the blood supply becomes insufficient and approximately 25 % of patients experience necrosis. When the cecum is viable, cecostomy, cecopexy, or resection is acceptable depending on the condition of the patient. Bowel resection is obviously required when the cecum has ischemic or necrotic changes.

Cecopexy involves untwisting of the cecum and fixation with sutures or the use of a peritoneal flap, but it has a high recurrence rate of up to 20 % [13]. Cecopexy may be selected in patients with a poor general condition. Intraoperative detorsion alone is associated with a failure rate ranging from 13 to 75 % [14].

Resection of involved intestinal segments that have ischemic change is required when the patient’s condition is stable. There are no reports of recurrence after resection. Primary anastomosis is acceptable when the transverse colon and ileum are viable.

Laparoscopic surgery for acute abdomen has diagnostic and therapeutic benefits [15]. These benefits involve the identification of the causes of acute abdomen and location of diseases in the intra-abdominal cavity. Entering the abdomen laparoscopically can be difficult because of the distended small bowel occupying the abdominal space. Experience in the laparoscopic technique is required to explore the abdominal cavity and handle adhesions, dilated intestines, and fluid collection. However, a laparoscopic approach can be attempted in selected cases of bowel obstruction [15].

Laparoscopic surgery for acute cecal volvulus is not widely reported, probably because undergoing a laparoscopic operation in these situations is uncommon. However, several cases of cecal volvulus have been treated with multi-port laparoscopic cecopexy and colectomy [16–18]. Handling the twisted cecum intraperitoneally until decompression is achieved is not feasible or safe because the cecum is tense and dilated. Aspiration and decompression are effective for laparoscopic ileus surgery.

In this report, we describe a method in which a Nelaton catheter was inserted into the dilated cecum through the appendix under direct vision, followed by laparoscopic mobilization of the appendix. This method is mainly used in open surgery for left-side obstructive colon cancer [5]. We introduced this method to single-site laparoscopic surgery, taking advantage of the appendix, which is easily extracted through an umbilical incision. An alternative method using transabdominal aspiration with a 25-G spinal needle was reported in 2008 by Schlinkert [18]. They performed a multiple-port laparoscopic right hemicolectomy, followed by decompression for acute cecal volvulus. However, to the best of our knowledge, there are no reports in the English literature regarding single-site laparoscopic colectomy for acute cecal volvulus. We searched for related reports in PubMed using the keywords “cecal volvulus,” “single,” and “laparoscopic surgery.”

Single-site laparoscopic colectomy is a recent technique in the field of laparoscopic surgery. All surgical instruments are placed through a small single incision, usually located at the umbilicus. This technique minimizes surgical invasiveness. Therefore, advantages of this technique include less pain, faster recovery, decreased incisional morbidity, and improved cosmesis compared with conventional techniques. Single-site laparoscopic colectomy is becoming standard practice for colon cancer and inflammatory bowel disease. Recently, for colon cancer, complete mesocolic excision with central vascular ligation was performed for radical oncological resection, even in single-site laparoscopic colectomy [19]. At our institute, this procedure is also routinely performed for elective right-sided colon cancer. The single-site laparoscopic technique was also smoothly applied to our case. Because the dilated colon is decompressed, untwisting it to a normal position laparoscopically and performing single-site laparoscopic colectomy are feasible.

## Conclusions

This is the first report of single-site laparoscopic right hemicolectomy that was adapted for acute cecal volvulus. If decompression is performed by catheterization of the cecum through the appendix followed by laparoscopic mobilization of the appendix, single-site laparoscopic colectomy can be safely and securely performed for acute cecal volvulus.
